# Characterisation of P2Y_12_ Receptor Responsiveness to Cysteinyl Leukotrienes

**DOI:** 10.1371/journal.pone.0058305

**Published:** 2013-03-05

**Authors:** Holly R. Foster, Elisabeth Fuerst, Tak H. Lee, Davis J. Cousins, Grzegorz Woszczek

**Affiliations:** MRC and Asthma UK Centre in Allergic Mechanisms of Asthma, Department of Asthma, Allergy and Respiratory Science, King’s College London, London, United Kingdom; Fundação Oswaldo Cruz, Brazil

## Abstract

Leukotriene E_4_ (LTE_4_), the most stable of the cysteinyl leukotrienes (cysLTs), binds poorly to classical type 1 and 2 cysLT receptors although in asthmatic individuals it may potently induce bronchial constriction, airway hyperresponsiveness and inflammatory cell influx to the lung. A recent study has suggested that the purinergic receptor P2Y_12_ is required for LTE_4_ mediated pulmonary inflammation in a mouse model of asthma and signals in response to cysLTs. The aim of the study was to characterise the responsiveness of human P2Y_12_ to cysteinyl leukotrienes. Models of human CysLT_1_, CysLT_2_ and P2Y_12_ overexpressed in HEK293, CHO cells and human platelets were used and responsiveness to different agonists was measured using intracellular calcium, cAMP and β-arrestin recruitment assays. CysLTs induced concentration dependent calcium mobilisation in cells overexpressing CysLT_1_ and CysLT_2_ but failed to induce any calcium response in cells expressing P2Y_12_ or P2Y_12_+ Gα_16_. In contrast, selective P2Y_12_ agonists ADP and 2-MeS-ADP induced specific calcium flux in cells expressing P2Y_12_+ Gα_16_. Similarly, specific response to 2-MeS-ADP, but not to cysLTs was also observed in cells expressing P2Y_12_ when intracellular cAMP and β-arrestin signalling were analysed. Platelets were used as a model of human primary cells expressing P2Y_12_ to analyse potential signalling and cell activation through P2Y_12_ receptor or receptor heterodimers but no specific LTE_4_ responses were observed. These results show that LTE_4_ as well as other cysLTs do not activate intracellular signalling acting through P2Y_12_ and suggest that another LTE_4_ specific receptor has yet to be identified.

## Introduction

Cysteinyl leukotrienes (cysLTs) (LTC_4_, LTD_4_, LTE_4_) are an important class of proinflammatory lipid molecules that are thought to mediate many of the principal features of bronchial asthma such as bronchial constriction, airway hyperresponsiveness and leukocyte trafficking. They are synthesized *in vivo* by immunocompetent cells such as mast cells, eosinophils, basophils and monocytes/macrophages [1]. Upon cell activation, intracellular phospholipase A_2_ releases arachidonic acid from membrane phospholipids. 5-lipoxygenase subsequently converts arachidonic acid to the unstable intermediate LTA_4_, which is then conjugated to reduced glutathione by leukotriene C_4_ synthase to form LTC_4_. After transport to extracellular space, LTC_4_ is converted to LTD_4_ and then to the terminal product LTE_4_, the most stable and the most abundant cysLT in biological fluids.

The biological actions of cysLTs are mediated by two currently identified G-protein coupled receptors (GPCR): CysLT type 1 receptor (CysLT_1_) and type 2 receptor (CysLT_2_). They differ in binding affinities for different cysLTs. CysLT_1_ is recognised as a high-affinity receptor for LTD_4_, whereas CysLT_2_ binds LTC_4_ and LTD_4_ with similar affinity [2], [3], [4], [5]. LTE_4_ has long been believed to be the final and least active metabolite of cysLTs, with low affinity for binding to the classical receptors and lowest functional agonistic potency in comparison to LTC_4_ and LTD_4_ [6].

Inhaled LTD_4_ has been shown to elevate the numbers of sputum eosinophils in subjects with asthma [7]. However, it was LTE_4_ that was shown to be the most potent cysLT in eliciting influx of inflammatory cells such as eosinophils and basophils into bronchial mucosa of asthmatic subjects [8], [9]. These preferential agonist functions of LTE_4_ were not explained by pharmacological properties of CysLT_1_ or CysLT_2_. Similarly, there is still no explanation for the comparable potency of LTE_4_ in comparison to LTC_4_ and LTD_4_ in eliciting a dermal wheal and flare reaction in human skin [10] and for equally effective contraction of human bronchi *in vitro* by all cysLTs [11]. All of the above data strongly suggests the existence of one or more specific LTE_4_ receptors that have not been identified to date. The potential existence of such a receptor has been recently demonstrated [12]. In a knock-out murine model, vascular permeability induced by intradermal injection of LTE_4_ in mice lacking both CysLT_1_ and CysLT_2_ exceeded the response to LTC_4_ and LTD_4_, suggesting the presence of another cysLT receptor that responds preferentially to LTE_4_. LTE_4_ was 64-fold more potent in CysLT_1_/CysLT_2_ double deficient mice than in wild type mice, revealing an inhibitory negative regulation of the novel LTE_4_ receptor by the two known receptors. It was also found that the pre-treatment of double deficient mice with a selective CysLT_1_ antagonist did not inhibit, but increased even further the permeability response to all cysLTs.

Interestingly, recent *in silico* modelling and *in vitro* studies suggested that LTE_4_ may be a ligand for an ADP receptor, P2Y_12_ when heterologously expressed as a fusion protein with human Gα_16_ [13]. Further evidence for the interaction of LTE_4_ with the purinergic receptor P2Y_12_ was provided by Paruchuri *et al.* who showed that P2Y_12_ was required for LTE_4_-mediated pulmonary inflammation [14]. Mice lacking the classical cysLT receptors maintained LTE_4_-induced eosinophilia, goblet cell metaplasia and IL-13 expression in response to low-dose of aerosolized allergen but P2Y_12_ knock-out and platelet-depleted mice (a cell type highly expressing P2Y_12_) showed a substantial loss in those functions. Although direct binding of labelled LTE_4_ to P2Y_12_ could not be demonstrated, data from cells overexpressing P2Y_12_ indicated that the presence of P2Y_12_ is required for signalling and activation by LTE_4_.

Human P2Y_12_ has been cloned and characterised with ADP as its natural agonist [15]. It has been shown to couple to Gα_i_ and signal in response to ADP by inhibiting adenylate cyclase activity and cAMP generation in human platelets and when heterologously expressed [16]. To address the question whether LTE_4_ is also a direct agonist for human P2Y_12_ or activates P2Y_12_ signalling through GPCR heterodimer interactions we characterised responsiveness to cysLTs in recombinant models of cell overexpressing P2Y_12_ and in platelets, primary human cells constitutively expressing P2Y_12_.

## Materials and Methods

### Reagents

Leukotrienes (LTC_4_, LTD_4_ and LTE_4_) were purchased from Cayman Chemical (Ann Arbor, Mich). ADP, 2-methylthio-adenosine-5′-diphosphate (2MeS-ADP), prostaglandin E_1_ (PGE_1_), isoproterenol, forskolin, 3-Isobutyl-1-methylxanthine (IBMX) and calcium ionophore (A23187) were purchased from Sigma-Aldrich (Dorset, UK).

### Cell Culture

HEK293 cells were cultured in high glucose (4500 mg/L) DMEM supplemented with 2 mmol/L glutamine, 10% fetal bovine serum and Penicillin/Streptomycin (50 units/ml/50 µg/ml) (all Life Technologies, UK) in a humidified 5% CO_2_ 37°C incubator. Cells were passaged every 3–4 days replacing all medium with fresh cell culture medium.

### Transient Transfection of HEK293 Cells

HEK293 cells cultured to above 60% confluence were transiently transfected with a mixture of Lipofectamine 2000 (Life Technologies) and the following plasmids as indicated: pcDNA3.1-human CysLT_1_, pcDNA3.1-human CysLT_2_, pcDNA3.1-human P2Y_12_, pcDNA3.1-3xHA human P2Y_12_, pcDNA3.1-human Gα_16_, pcDNA3.1-3xHA human ADRB2 (all the Missouri S&T cDNA Resource Center, Rolla, Mo) and pCMV6-Kan/Neo- mouse P2Y_12_ (Origene Technologies) in serum-free medium (Opti-MEM, Life Technologies) according to manufacturer’s protocol. After incubation the transfection medium was removed and HEK293 cells were cultured for 36 hours in standard culture medium at 37°C in a humidified 5% CO_2_ incubator.

### Preparation of Platelet-rich Plasma

The study was approved by the Research Ethics Committee of Guy’s Hospital. Blood was collected over citrate-dextrose solution (ACD, 6∶1) from patients who had provided written informed consent prior to any procedure. Platelet-rich plasma (PRP) was prepared by centrifugation at 150 g for 15 minutes at room temperature. PRP was centrifuged for a further 5 minutes to reduce erythrocyte contamination.

### Calcium Mobilisation Assay

Calcium mobilisation assays were conducted using FLIPR calcium 4 assay kit (Molecular Devices, Sunnydale, CA) as described previously [17], [18]. HEK293 cells (1.5×10^5^/well) were plated into poly-D-lysine coated 96 well plates in RPMI 1640 supplemented with 10 mmol/L HEPES. After a 5-hour incubation, cells were incubated for 1 hour with FLIPR loading buffer prior to addition of ligand and fluorescent intensity was measured at 37°C using a Flexstation 3 (Molecular Devices). Controls included medium control with ethanol for leukotriene stimulations.

PRP was washed in modified Tyrode’s buffer (pH 6.2, 150 mol/L NaCl, 3 mmol/L KCl, 5 mmol/L glucose, 1 mmol/L MgCl_2_, 10 mmol/L HEPES, 0.1% BSA) supplemented with 0.5 µmol/L PGE_1_. Platelets (2×10^6^/well) were plated into 96-well plates in modified Tyrode’s buffer supplemented with 1.26 mmol/L CaCl_2_ and incubated for 30 minutes with FLIPR loading buffer supplemented with 2.5 mmol/L probenecid and fluorescence was measured using a Flexstation 3. Results were analysed with SoftMax Pro Software (Molecular Devices).

### Analysis of Receptor Surface Expression

Washed transfected HEK293 cells were stained with an Alexa Fluor 488 conjugated anti-HA monoclonal antibody (Clone 16B12, Covance, Ca) that recognises the HA epitope that is N-terminally located on the receptors of interest. Analysis was performed on a FACScalibur with CellQuest Pro software (BD Biosciences).

### cAMP Accumulation Assay

Intracellular cAMP accumulation was analysed in HEK293 cells using the Cyclic AMP assay kit (Meso Scale Discovery, Gaithersburg, MD, USA) following manufacturer’s protocols. HEK293 cells with added IBMX (1 µmol/L) were plated on anti-cAMP coated MULTI-ARRAY 96-well small spot plates (MSD), stimulated with forskolin and agonists for 15 minutes as indicated, lysed and run on the ImageSector 6000 (Meso Scale Discovery). Results were analysed using MSD workbench software.

Intracellular cAMP accumulation was analysed in PRP using the HitHunter® cAMP XS+ assay kit (DiscoverX, UK) following manufacturer’s protocols. PRP was centrifuged at 1000 g for 10 minutes and washed three times with pre-chilled DPBS supplemented with 2 mmol/L EDTA. Platelets (5×10^6^/well) suspended in DPBS supplemented with 2 mmol/L EDTA and 1 µmol/L IBMX, were plated on a 96-well plate, stimulated with forskolin and other agonists for 20 minutes as indicated and incubated with detection reagents. Luminescent signal was measured 4 hours after lysis using a Flexstation 3. Results were analysed with SoftMax Pro Software.

### β-arrestin Recruitment Assay

Analysis of β-arrestin recruitment was conducted using a Pathhunter eXpress β-arrestin kit (DiscoverX) following manufacturer’s protocols. In this system, the GPCR and β-arrestin are fused to two fragments of β-galactosidase and the interaction of the two proteins results in an enzymatic reaction. In brief, CHO cells stably transfected with the C-terminally modified human (mouse) P2Y_12_ and with the β-arrestin, N-terminally tagged with deletion mutant of β-galactosidase, were seeded on 96-well plates in OCC medium for a 48 hour recovery period at 37°C. Cells were stimulated for 90 minutes at 37°C as indicated and then incubated with detection reagents for a further 90 minutes at room temperature. Luminescent signal, which is directly related to the recruitment of β-arrestin to P2Y_12_ in the assay, was measured using a Flexstation 3.

### Analysis of Platelet Activation by Flow Cytometry

Whole peripheral blood drawn over ACD (6∶1) was stimulated immediately after collection for 10 minutes at room temperature. 5 µl of blood was directly stained with monoclonal antibodies against CD61 (APC, clone VI-PL2) and CD62P (PE, clone Psel.KO2.3) or appropriate isotype controls (all eBiosciences). Cells gated in the platelet population were analysed for platelet activation using a FACScalibur with CellQuest Pro software (BD Biosciences).

### CCL5/RANTES ELISA

PRP supplemented with 0.5 µmol/L PGE_1_ was centrifuged at 1000 g for 10 minutes and washed three times with pre-warmed modified Tyrode’s buffer supplemented with 0.5 µmol/L PGE_1_. PRP was stimulated for 15 minutes with indicated agonists and CCL5 was measured in supernatants using a CCL5/RANTES duo set kit (R&D Systems, UK) following manufacturer’s protocol. Optical density was recorded on Anthos htIII (Anthos Labtech) using Stingray (DazDaq) software. Measurements at 450 nm were corrected by measurement at 578 nm and concentrations of RANTES/CCL5 were generated from the standard curve.

### Statistical Analysis

Data were analysed by means of one- or two- way ANOVA using GraphPad Prism software (GraphPad, La Jolla, Ca). Differences were considered significant at a p-value of less than 0.05.

## Results

### CysLT-mediated Calcium Mobilisation in Models of Transiently Expressed CysLT_1_, CysLT_2_ and P2Y_12_

**Figure 1:**
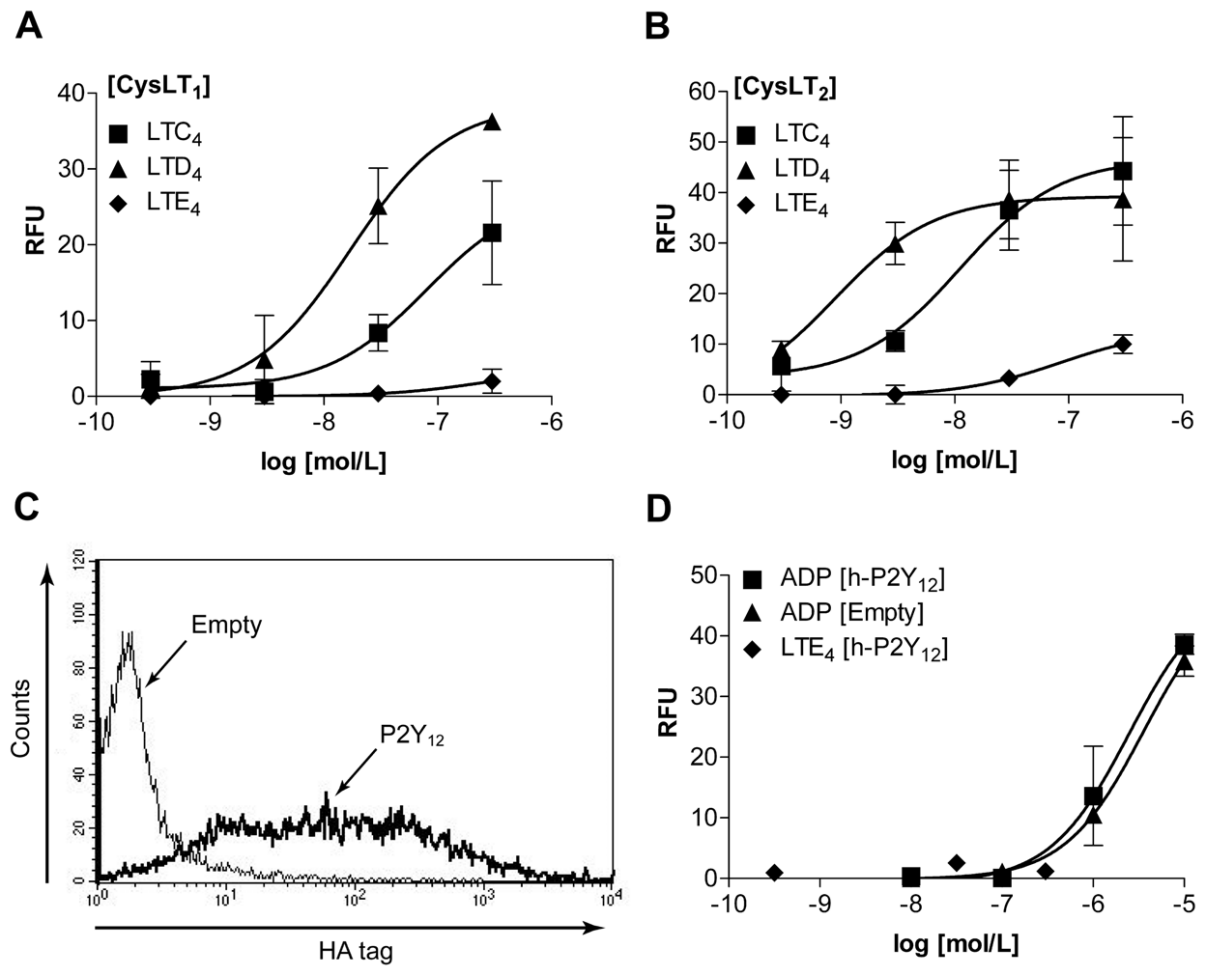
Effect of LTE_4_ on calcium mobilisation in h-P2Y_12_ overexpression model. HEK293 cells were transiently transfected with indicated vectors and intracellular responses recorded. (A) [h-CysLT_1_] and (B) [h-CysLT_2_] transfectants stimulated with indicated concentrations of LTC_4_, LTD_4_ and LTE_4_. N = 6. (C) Flow cytometry analysis of h-P2Y_12_ protein surface expression in [h-P2Y_12_] and [Empty] transfectants using an anti-HA antibody, representative of three experiments. (D) [h-P2Y_12_] and [Empty] transfectants stimulated with ADP or LTE_4_, N = 9. Results (A), (B) and (D) represented as peak intracellular calcium response, relative fluorescence units (RFU), mean ± S.E.M.

To ascertain whether LTE_4_ could mediate signal transduction through the P2Y_12_ receptor, a model of heterologous receptor expression was established in HEK293 cells. These cells do not natively express any known classical cysLT receptors or P2Y_12_ and when unmodified they do not respond to cysLT stimulation. To validate this model, constructs expressing human (h)-CysLT_1_ and h-CysLT_2_ were transiently transfected into separate HEK293 populations as previously described [5]. The transfectants were stimulated with exogenous LTC_4_, LTD_4_ and LTE_4_ and their intracellular calcium responses were measured using FLIPR Calcium 4 assay kit and a FlexStation 3, showing similar receptor potency as reported previously, with LTE_4_ having the lowest potency for calcium mobilisation in comparison to LTC_4_ and LTD_4_. (Fig. 1A–B) [19]. Constructs expressing h-P2Y_12_ with N-terminal 3xHA tag were then transiently transfected into HEK293 cells and surface expression was verified by flow cytometry (Fig. 1C). On stimulation with exogenous cysLTs or ADP, the known natural ligand for h-P2Y_12_, no difference was found in intracellular calcium flux responses between the h-P2Y_12_ transfectants and controls transfected with empty vectors (Fig. 1D), although calcium response to ADP was observed reflecting constitutive expression of other purinergic receptors and showing that h-P2Y_12_ signal transduction does not occur through calcium mobilisation in this model.

### LTE_4_-mediated Calcium Mobilisation in Gα_16_ co-transfection Models

**Figure 2:**
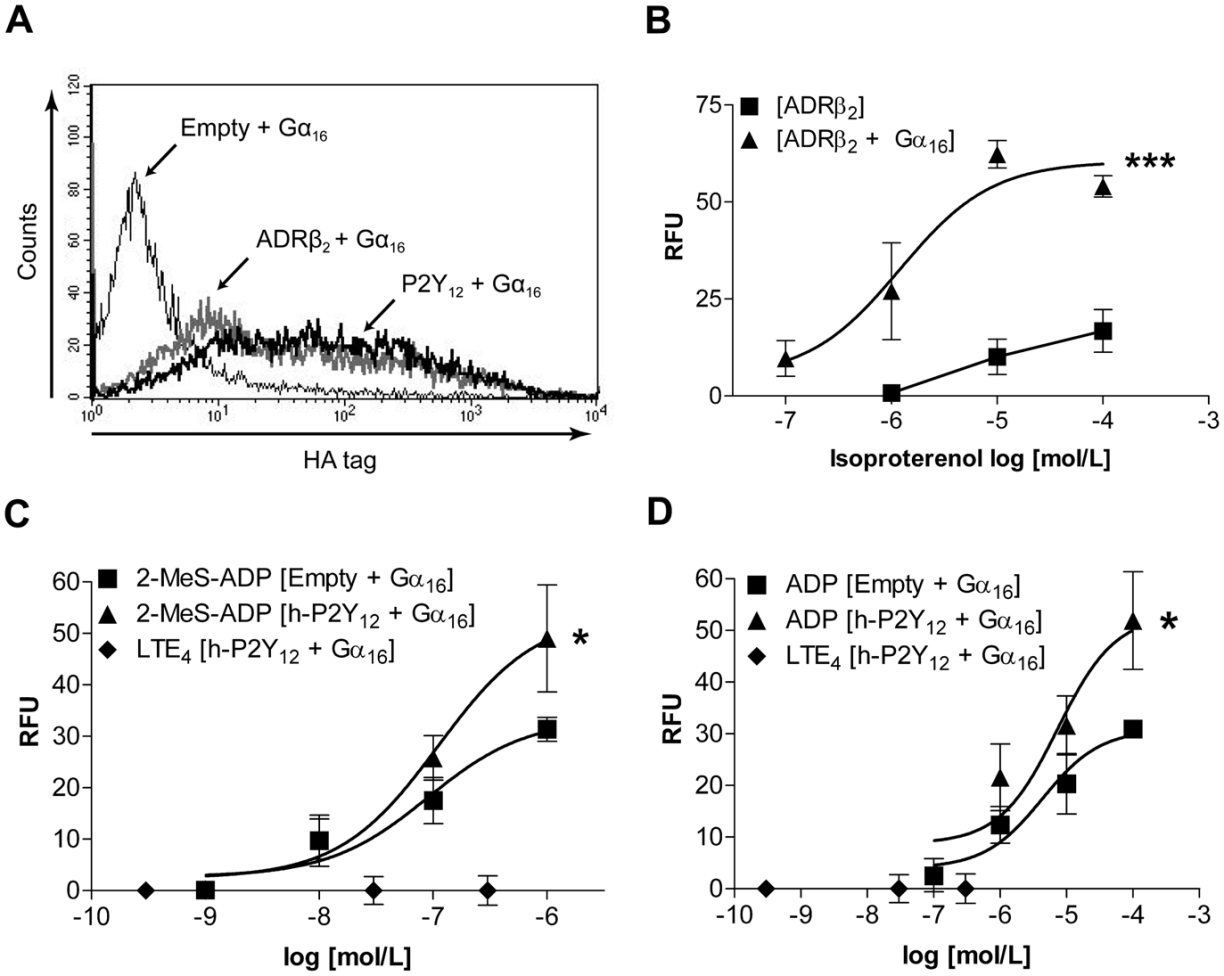
Effect of LTE_4_ on HEK293 cells co-transfected with h-P2Y_12_ and G_α16_. HEK293 cells were transiently transfected with vectors indicated and intracellular responses recorded. (A) Flow cytometry analysis of ADRβ_2_ and h-P2Y_12_ protein surface expression in [ADRβ_2_+ G_α16_] and [hP2Y_12_+ G_α16_] transfectants respectively, representative of three experiments. ADRβ_2_ and h-P2Y_12_ were detected using an anti-HA antibody, representative of three experiments. (B) [ADRβ_2_] and [ADRβ_2_+ G_α16_] transfectants stimulated with isoproterenol, N = 6, 2-way ANOVA p = 0.0003. [h-P2Y_12_+ G_α16_] and [Empty+G_α16_] transfectants stimulated with LTE_4_ and either (C) 2-MeS-ADP or (D) ADP, N = 9. 2-way ANOVA between (C) 2-MeS-ADP responses p = 0.0190 and (D) ADP responses p = 0.0220. Results (B), (C) and (D) presented as peak intracellular calcium response mean ± S.E.M.

Co-transfections of G-protein coupled receptors (GPCRs) and the fusion protein, Gα_16_, have been reported previously to directly activate phospholipase C and calcium signalling [20]. To demonstrate the effectiveness of this approach in our recombinant model we overexpressed human β_2_ adrenergic (h-ADRβ_2_) receptor and stimulated with isoproterenol. A construct encoding h-ADRβ_2_ with N-terminal 3xHA tag was transiently co-transfected with a construct containing h-Gα_16_ into HEK293 cells and surface expression of the receptor was verified by flow cytometry (Fig. 2A). On stimulation with exogenous isoproterenol, h-ADRβ_2_ transfectants overexpressing h-Gα_16_ were able to produce a statistically significant (p = 0.0003; 2-way ANOVA) increase in calcium flux compared to h-ADRβ_2_ transfectants showing that h-Gα_16_ has the potential to modulate GPCR signal transduction in our HEK293 heterologous expression model (Fig. 2B).

Constructs containing tagged h-P2Y_12_ were then co-transfected with h-Gα_16_ and receptor surface expression was confirmed by flow cytometry (Fig. 2A). These transfectants were also able to show statistically significant increases in calcium responses to ADP (p = 0.022; 2-way ANOVA) and its more stable derivative, 2-MeS-ADP (p = 0.019; 2-way ANOVA), compared to control transfectants (fig. 2C–D). Negligible increases in calcium flux were observed upon LTE_4_ stimulation of the h-P2Y_12_+ h-G_α16_ transfectants (Fig. 2C–D) and upon stimulation with LTC_4_ and LTD_4_ (data not shown).

### P2Y_12_-induced Intracellular cAMP Signalling

**Figure 3:**
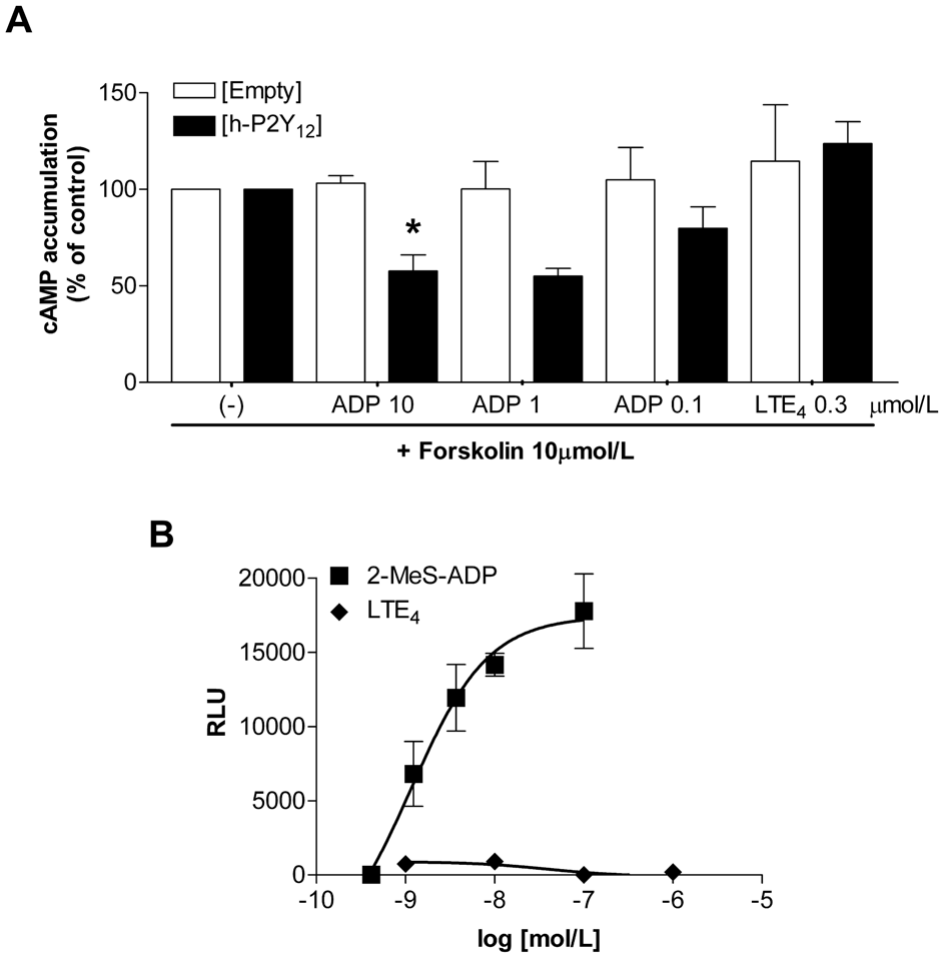
Effect of LTE_4_ stimulation on cAMP and β-arrestin signalling pathways. Intracellular cAMP concentrations and β-arrestin recruitment was analysed in models of transiently transfected HEK293 or stably modified CHO cells, respectively. (A) [h-P2Y_12_] and [Empty] transfectants were stimulated with forskolin and either ADP or LTE_4_, N = 6, data expressed as % of forskolin stimulated control. (B) CHO cells expressing h-P2Y_12_ and β-arrestin were stimulated with either 2-MeS-ADP or LTE_4_, N = 9, expressed as relative luminescence units (RLU). Data represented as mean ± S.E.M. Two-way ANOVA with Bonferroni post-hoc test, *p<0.05.

Human P2Y_12_ has been shown to signal physiologically through Gα_i_ and by inhibition of intracellular cAMP generation. To analyse this potential signalling pathway, h-P2Y_12_ transfectants were stimulated with forskolin to activate adenylyl cyclase and to increase cAMP levels, together with ADP or cysLTs and intracellular cAMP was measured using a competitive immunoassay. ADP induced a significant, concentration dependent inhibition of forskolin induced cAMP in h-P2Y_12_ transfectants, while LTE_4_ treatment showed no statistically significant difference in cAMP accumulation over a range of 0.3–300 nmol/L concentrations, from the empty vector transfectants (Fig. 3A). This confirms that h-P2Y_12_ does not signal directly through the coupling of either to Gα_q_ or Gα_i_ upon LTE_4_ stimulation. We could also exclude Gα_s_ signalling in our model as no change in cAMP levels was observed when h-P2Y_12_ transfected cells were stimulated with ADP or cysLTs alone (not shown).

### P2Y_12_-induced β-arrestin Signalling

Recruitment of β-arrestin is another activation event that can be analysed as a measure of GPCR activation. Until recently, an agonist’s efficacy for β-arrestin recruitment was believed to be proportional to its efficacy for G-protein activities. However, it has been demonstrated that “biased ligands” can selectively activate β-arrestin function and elicit specific biological effects [21]. To address the question of β-arrestin specific signalling induced by LTE_4_, C-terminally modified h-P2Y_12_ stably transfected into CHO cells with β-arrestin N-terminally tagged with a deletion mutant of β-galactosidase were stimulated with either 2-MeS-ADP or cysLTs and processed according to manufacturer’s protocol (Fig. 3B). Stimulation with 2-MeS-ADP induced a concentration dependent luminescent signal relating to the recruitment of β-arrestin to the h-P2Y_12_ receptor. Stimulation with LTE_4_ and other cysLTs showed no significant increase in signal suggesting a lack of β-arrestin pathway activation by leukotrienes. Collectively these observations show that cysLTs do not induce G-protein dependent or independent signalling pathways directly through h-P2Y_12_ indicating that h-P2Y_12_ is not a cysLT receptor.

### LTE_4_-mediated Signalling through Mouse P2Y_12_

**Figure 4:**
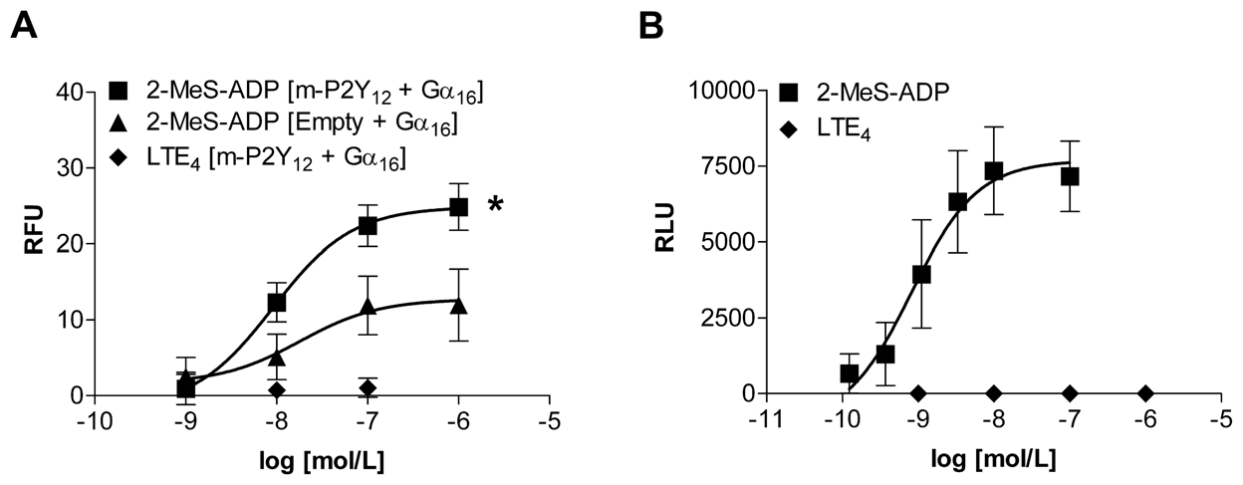
Effect of LTE_4_ stimulation on calcium and β-arrestin signalling pathways in mouse P2Y_12_ transfectants. HEK293 cells were transiently transfected with indicated vectors and intracellular calcium responses recorded. (A) [m-P2Y_12_+ G_α16_] and [Empty+G_α16_] transfectants were stimulated with LTE_4_ and 2-MeS-ADP, N = 9, 2-way ANOVA between 2-MeS-ADP responses p = 0.0101. (B) CHO cells stably expressing m-P2Y_12_ and β-arrestin were stimulated with either 2-MeS-ADP or LTE_4_, N = 9. Data presented as mean ± S.E.M.

The ability of LTE_4_ to mediate pulmonary inflammation has been shown to be dependent on P2Y_12_ expression in mouse models, as indicated by Paruchuri *et al.* [14]. Strong evidence shows that on removal of the P2Y_12_ receptor, either by knock down or by platelet depletion, the influx of inflammatory cells to the mouse lung can be significantly diminished. Although these findings are contradictory to our results, the lack of LTE_4_ mediated signalling in our recombinant model could be due to species differences as mouse (m)-P2Y_12_ shares only 89% homology to its human derivative [22]. To test this possibility a construct encoding m-P2Y_12_ were transiently transfected into HEK293 cells and stimulated with exogenous 2-MeS-ADP, LTC_4_, LTD_4_ or LTE_4_. Although a robust calcium flux was detected in response to 2-MeS-ADP stimulation, it did not differ in comparison to empty vector control transfectants and no response, similar to that of the human P2Y_12_ (Fig. 1D), was recorded for cysLT stimulation (data not shown). Co-transfections of m-P2Y_12_ and Gα_16_ were then employed to direct any signal transduction to activate calcium mobilisation. 2-MeS-ADP stimulation of these co-transfectants was able to induce a statistically significant increase in calcium mobilisation compared to the control transfectants (Fig. 4A) indicating that h-Gα_16_ is sufficiently able to direct the signalling pathway of m-P2Y_12_. No specific calcium flux was observed on stimulation of these co-transfectants with LTE_4_ (Fig. 4A).

To determine whether LTE_4_ could activate m-P2Y_12_ via the recruitment of β-arrestin, stable CHO cell transfectants of C-terminally modified m-P2Y_12_ and N-terminally tagged β-arrestin were stimulated with either 2-MeS-ADP or cysLT. Although stimulation with 2-MeS-ADP induced a dose-dependent luminescent signal, LTE_4_ stimulation produced negligible effects (Fig. 4B). These results show that m-P2Y_12_ signalling responses towards cysLT stimulation are similar to that of h-P2Y_12_.

### LTE_4_-induced Signalling and Cell Activation in Human Platelets

**Figure 5:**
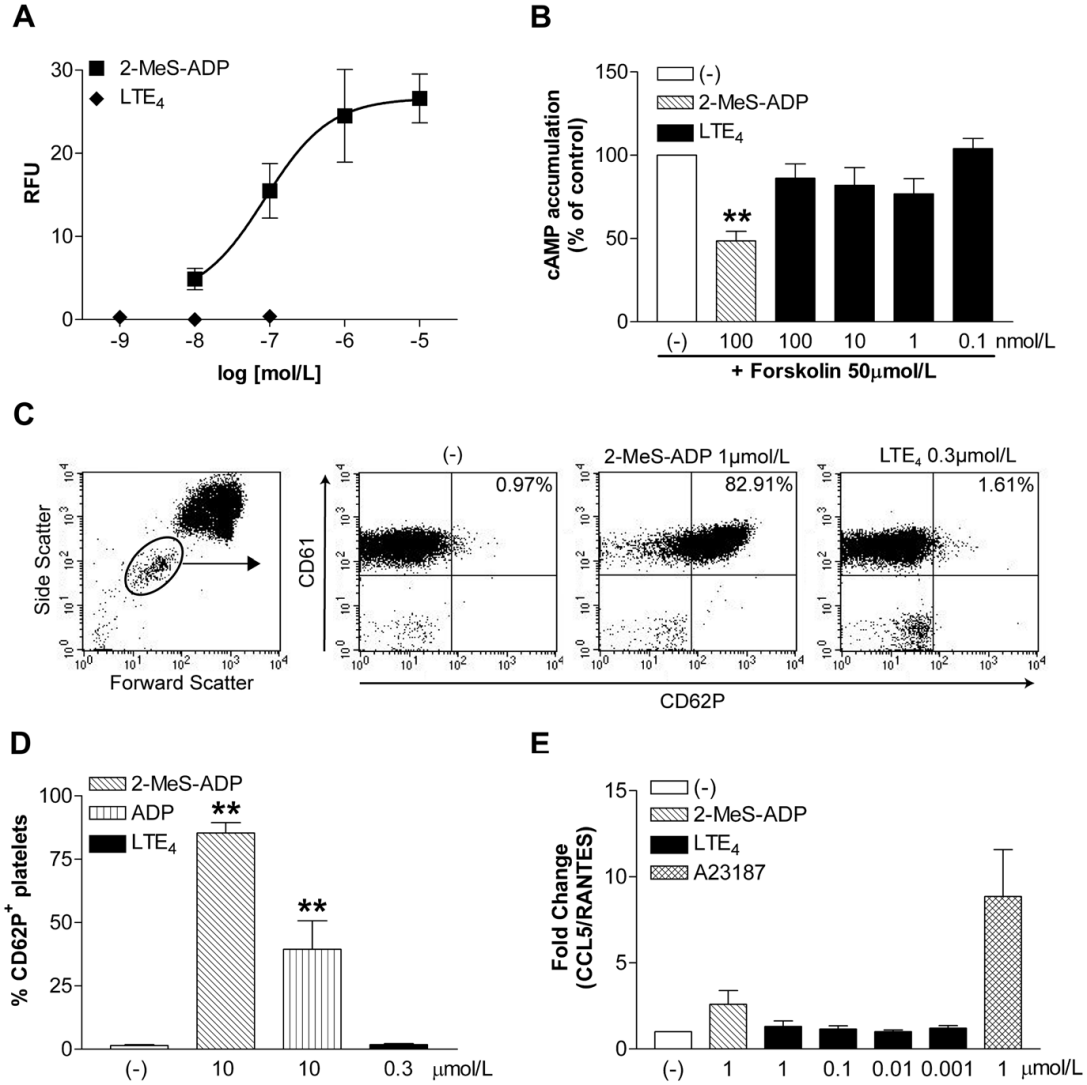
The effect of LTE_4_ stimulation on human platelets. Isolated human platelets were (A) stimulated with 2-MeS-ADP and LTE_4_ and peak intracellular calcium mobilisation responses were recorded, data from 3 donors run in duplicate, (B) stimulated with forskolin and either LTE_4_ or 2-MeS-ADP for 15 minutes in the presence of IBMX, data from 3–6 donors run in triplicate, expressed as % of forskolin stimulated control. (C) Whole blood stimulated with either 2-MeS-ADP or LTE_4_ for 10 minutes was stained with monoclonal antibodies for CD61 and CD62P. Flow cytometry analysis of platelet activation was performed on gated platelet population, representative of three experiments. (D) Flow cytometry analysis of platelet activation, data presented as mean ± S.E.M of 3 experiments with different donors. (E) Isolated human platelets were stimulated with either LTE_4_, 2-MeS-ADP or calcium ionophore, supernatants collected and CCL5 concentrations measured using ELISA. Data presented as mean ± S.E.M of 3 experiments with different donors run in duplicate. One-way ANOVA performed **p<0.01.

Physiologically, the signalling capability of P2Y_12_ is highly modulated by another ADP purinergic receptor, P2Y_1_ [23]. Signal modulation, whether this is through heterodimerisation or reciprocal cross-talk, allows the potentiation of signalling responses from both P2Y_12_ and P2Y_1_. To determine whether the importance of P2Y_12_ in LTE_4_ mediated pulmonary inflammation *in vivo* could be due to such interactions or heterodimerisation of GPCRs, isolated human platelets, one of very few cell types that highly expresses P2Y_12_, were stimulated with 2-MeS-ADP and LTE_4_ and their intracellular signalling responses were analysed. A robust calcium mobilisation in a dose dependent manner and a statistically significant inhibition of cAMP were generated by 2-MeS-ADP stimulation (Fig. 5A–B) indicating that platelets isolated from whole blood were functionally intact and are responsive to P2Y_12_ agonists. LTE_4_ stimulation was unable to generate any calcium mobilisation and no significant inhibition of cAMP was observed (Fig. 5A–B) which is in agreement with the recombinant model data (Fig. 1D and 3A).

Activated platelets enter into an aggregation cascade where adhesion molecules are upregulated on the cell surface and range of stored mediators are released to enhance this process. To address the question whether cysLTs are able to activate platelets through P2Y_12_ or physiologically expressed receptor heterodimers, platelet activation measured by expression of P-selectin (CD62P) and release of stored chemokine CCL5 (RANTES) were analysed. Whole blood was stimulated with either ADP, 2-MeS-ADP or cysLTs and CD62P expression was measured by flow cytometry on the CD61^+^ population of human platelets (Fig. 5C). Whereas a robust upregulation of CD62P was observed after ADP or 2-MeS-ADP stimulation, no specific response to LTE_4_ (Fig. 5C–D) or other cysLTs (data not shown) was observed. Similarly, as human platelets stimulated with calcium ionophore or ADP released increased amounts of CCL5, no such a response was identified upon stimulation with LTE_4_ (Fig. 5E) or other cysLTs (data not shown).

## Discussion

This study highlights the complexities in leukotriene receptor biology and leukotriene signalling pathways involved in the immune response in chronic inflammatory diseases, such as atherosclerosis, asthma and rhinosinusitis. Ever since the elucidation and cloning of the two human cysLT receptors, CysLT_1_ and CysLT_2_, LTE_4_ has become the forgotten mediator in cysLT biology [6]. Its apparent weak efficacy in recombinant systems, poor binding affinities compared to LTC_4_ and LTD_4_, and the availability of selective CysLT_1_ antagonists, sidelined LTE_4_ as an important target for basic and clinical research. Recent studies have re-examined the role of LTE_4_ in pulmonary inflammation in relation to P2Y_12_ (a receptor proposed as a putative LTE_4_ receptor) [13]. Low dose OVA-induced allergic lung inflammation murine models lacking P2Y_12_ functionality either by knock down, platelet depletion or receptor antagonism, exhibited a substantial reduction in LTE_4_ mediated pulmonary inflammation, a phenomenon not observed in CysLT_1_/CysLT_2_ double knock out mice [14]. We undertook this study to determine whether these observations were specifically related to direct P2Y_12_-LTE_4_ interactions, therefore determining whether P2Y_12_ was in fact a cysteinyl leukotriene receptor.

Firstly, we established the heterologous expression model in which the recombinant receptors of interest could be transiently overexpressed in HEK293 cells. The best understood cysteinyl leukotriene receptor signalling pathway couples to Gα_q_ thus functional validation of the expression model was carried out by analysing intracellular calcium mobilisation. The pattern of ligand efficacies in both CysLT_1_ and CysLT_2_ transfectants matched those observed in the literature (LTD_4_ being the most potent agonist and LTE_4_ being the weakest) (Fig. 1) and with CysLT_1_ responses being sensitive to the specific CysLT_1_ antagonist, MK-571 (data not shown), therefore validating the expression model. Unsurprisingly, calcium mobilisation was not induced by cysteinyl leukotriene stimulation of P2Y_12_ transfectants, as the purinergic receptor has been well characterised as a GPCR that mainly activates Gα_i_ signalling pathways, affecting intracellular cAMP levels. However, the lack of LTE_4_ calcium mobilisation in P2Y_12_ transfectants co-expressing the Gα_16_ protein was in stark contrast with the initial study by Nonaka *et al.* identifying LTE_4_ as a surrogate ligand for P2Y_12_ [13]. They showed that LTE_4_, but not LTC_4_ or LTD_4_, was able to induce intracellular calcium flux in CHO cells stably overexpressing both h-P2Y_12_ and Gα_16_. As different platforms for the recombinant overexpression model were utilised, as well as different transfection techniques, this could be a reason why different signalling responses were observed. Further confirmation of our observations was shown by the negligible effect of LTE_4_ and other cysteinyl leukotrienes on cAMP accumulation and β-arrestin recruitment (Fig. 3), two intracellular signalling pathways being potently activated in the same assays by known P2Y_12_ agonists, ADP and 2-MeS-ADP. This lack of LTE_4_ induced signalling was mimicked in transfectants containing the mouse version of the P2Y_12_ receptor (Fig. 4) suggesting that the deficiency in signalling responses was not merely a human phenomenon and was independent of species variation. As no direct P2Y_12_ signalling upon cysLTs stimulation was observed in any of our recombinant experiments, we decided to address another possibility that LTE_4_ activates cells through another GPCR forming a heterodimer with P2Y_12_ *in vivo*. Platelets are one of very few cell types that functionally express P2Y_12_ and platelet depletion potently inhibited LTE_4_ mediated pulmonary inflammation (9) so we used human platelets to verify whether those cells are able to respond to cysLTs. No specific responses to LTE_4_ or other leukotrienes were observed when intracellular signalling (calcium, cAMP) as well as cell activation (P-selectin expression and CCL5/RANTES release) was measured. In contrast, human platelets strongly responded to known P2Y_12_ agonists and non-specific activators in those assays showing that cells were able to respond to appropriate stimulations implying that platelets are not a direct target for leukotrienes.

If platelets and P2Y_12_ do not respond to LTE_4_ as our data suggests, a question arises how observations from Paruchuri *et al.* on LTE_4_ mediated pulmonary inflammation may be explained? Our hypothesis is that LTE_4_ must activate specific receptors present on cells other than platelets, potentially structural cells such as endothelial cells, smooth muscle cells or tissue resident cells i.e. mast cells. Upon LTE_4_ activation, such cells would produce (release) mediator(s) activating platelets or platelet-adherent leukocytes, facilitating cell adhesion to endothelium, cell activation and migration to tissue and as a result enhancing inflammatory responses. Platelet involvement in proinflammatory reactions, especially in pulmonary inflammation observed in asthma has been of increased interest recently. Clinical evidence has demonstrated increases in circulating platelets in atopic asthmatics, as well as increases in leukocyte-platelet aggregates after allergen challenge [24], [25], [26]. Recent advancements in the field have shown the direct importance of platelets in leukocyte recruitment and airway remodelling in allergic inflammation [14], [27], [28]. Therefore the reduction in LTE_4_ mediated pulmonary inflammation seen in the study by Paruchuri *et al.* could be directly due to loss of P2Y_12_ functionality rather than LTE_4_ specific phenomenon [14]. However the studies of Paruchuri *et al.* and Maekawa *et al.* have elegantly highlighted that LTE_4_ signalling can occur independently to the classical cysLT receptors, CysLT_1_ and CysLT_2_ [14], [29] proving that LTE_4_ preferentially signals via another as yet unidentified cysLT receptor.

In conclusion, our study strongly suggests that LTE_4_ does not activate signalling either solely through P2Y_12_ or through P2Y_12_ being modulated by another receptor. The requirement to discover the true receptor for LTE_4_ is still very apparent so that more effective anti-leukotriene therapies can be developed for the treatment of asthma.
